# Supplementary material for: Devil's staircase transition of the electronic structures in CeSb
Source: Nat Commun. 2020 Jun 8;11:2888. doi: 10.1038/s41467-020-16707-6 (PMC7280508; doi:10.1038/s41467-020-16707-6)
Supplement: Supplementary file 1 — Supplementary Information [file 41467_2020_16707_MOESM1_ESM.pdf]

Supplementary Information:

Devil's staircase transition of the electronic structures in CeSb

Kuroda *et al.*

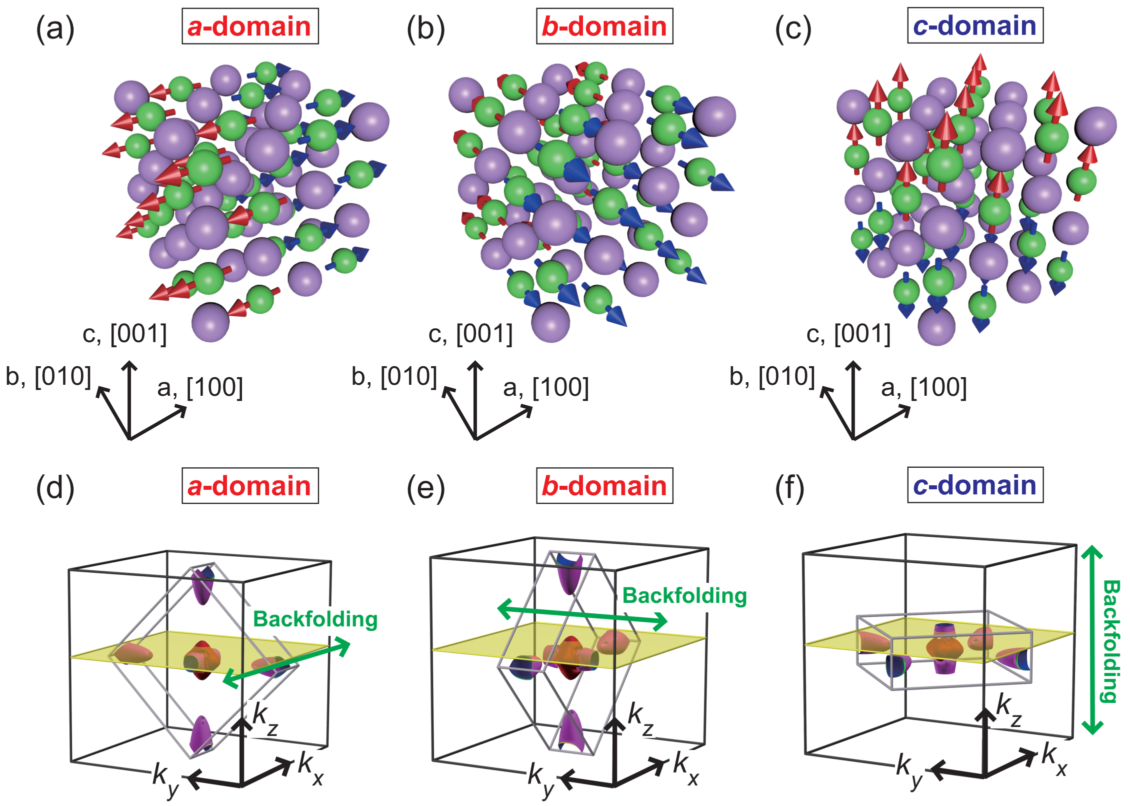

Supplementary Figure 1: **Definitions of the three magnetic domains with different orientations of 4f moment, and their reconstructed Fermi surfaces.** (a)-(c) The three magnetic domains with different orientation of the 4f moment along [100] (a-axis), [010] (b-axis) and [001] (c-axis). (d)-(f) The corresponding Fermi surfaces in the tetragonal Brillouin zone. Back-folding should occur along the 4f moment direction as well as the q direction. The yellow plane indicates the measured k_x-k_y plane with k_z=0.2Å^{-1} detected by photon energy of our laser 7 eV (see supplementary-Fig. 2).

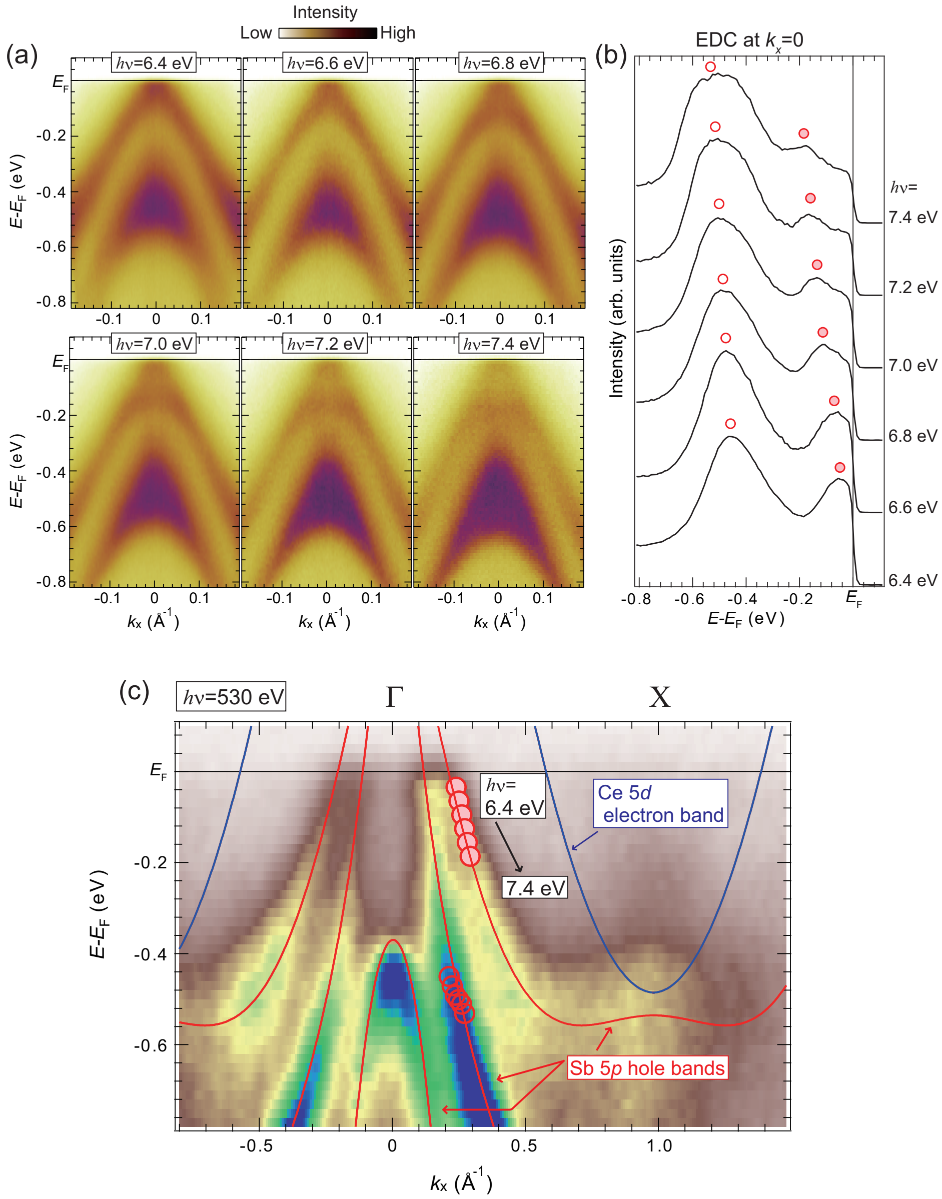

Supplementary Figure 2: k_z **dispersion of Sb 5**p **hole bands observed by synchrotron radiation (SR) ARPES.** (a) SR-ARPES images of Sb 5p hole bands at paramagnetic phase (T=60 K) obtained by using various photon energies ($h\nu$) from 6.4 eV to 7.4 eV. (b) Their energy distribution curves (EDCs) at k_x=0 Å^{-1}. The shape of the observed spectra varies with changing the $h\nu$ (closed and opened circles), displaying to the k_z dispersion. Thus, our ARPES by utilizing low-$h\nu$ (7 eV) light source sensitively detects the bulk electronic structures. (c) soft x-ray ARPES band maps with $h\nu$=530 eV cut along Γ-X line in paramagnetic phase [49], compared with DFT+U calculation (colored lines). The closed and opened circles indicate the k_z dispersions obtained by the $h\nu$ data in (a) and (b), which respectively correspond to the outer and inner hole bands at k_z ~0.2 Å^{-1}. By these systematic comparisons, we have determined that our laser with $h\nu$ of 7 eV mainly cuts the k_x-k_y plane at k_z ~0.2 Å^{-1}, almost corresponding to the k_F of the outer hole band. Correspondingly, as shown in Figs. **3d** and **3g**, our laser-ARPES observes only its tiny hole pocket at E_F, consistent with the DFT+U calculation (Figs. **3a** and **3j**). The blurred photoelectron intensities outside the main signal in Fig. **3d** come from the signals from the other k_z due to the k_z broadening effect [1, 2].

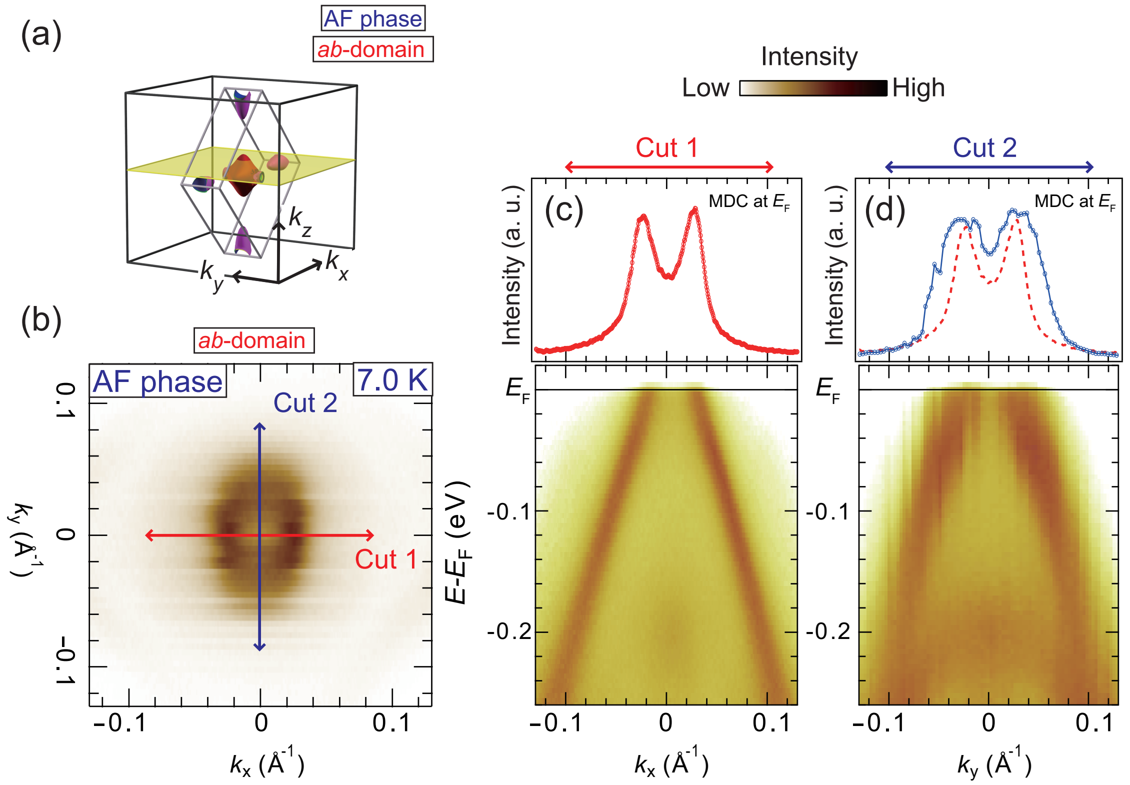

Supplementary Figure 3: **Negligible anisotropy in the observed Fermi surface for *ab*-domain by laser-ARPES.** (a) The reconstructed FS of AF phase for *ab*-domain. The colored $k_x - k_y$ plane corresponds to the momentum sheets at k_z=0.2 Å^{-1} cut by our laser-ARPES. (b) The result of the Fermi surface mapping in AF phase (T=7.0 K) for *ab*-domain, corresponding to Fig. 3**e** in the main text. (c), (d) the E-k maps along cut1 (k_x) and cut2 (k_y), and their momentum distribution curves (MDCs) at E_F. In (d), the MDC along k_y is compared to that along k_x as denoted by the dashed line. The deviation between the k_F for the both momentum cuts is found to be negligibly small, which thus indicates that the anisotropy in the tetragonal Fermi surface is rather weak at the measured k_z.

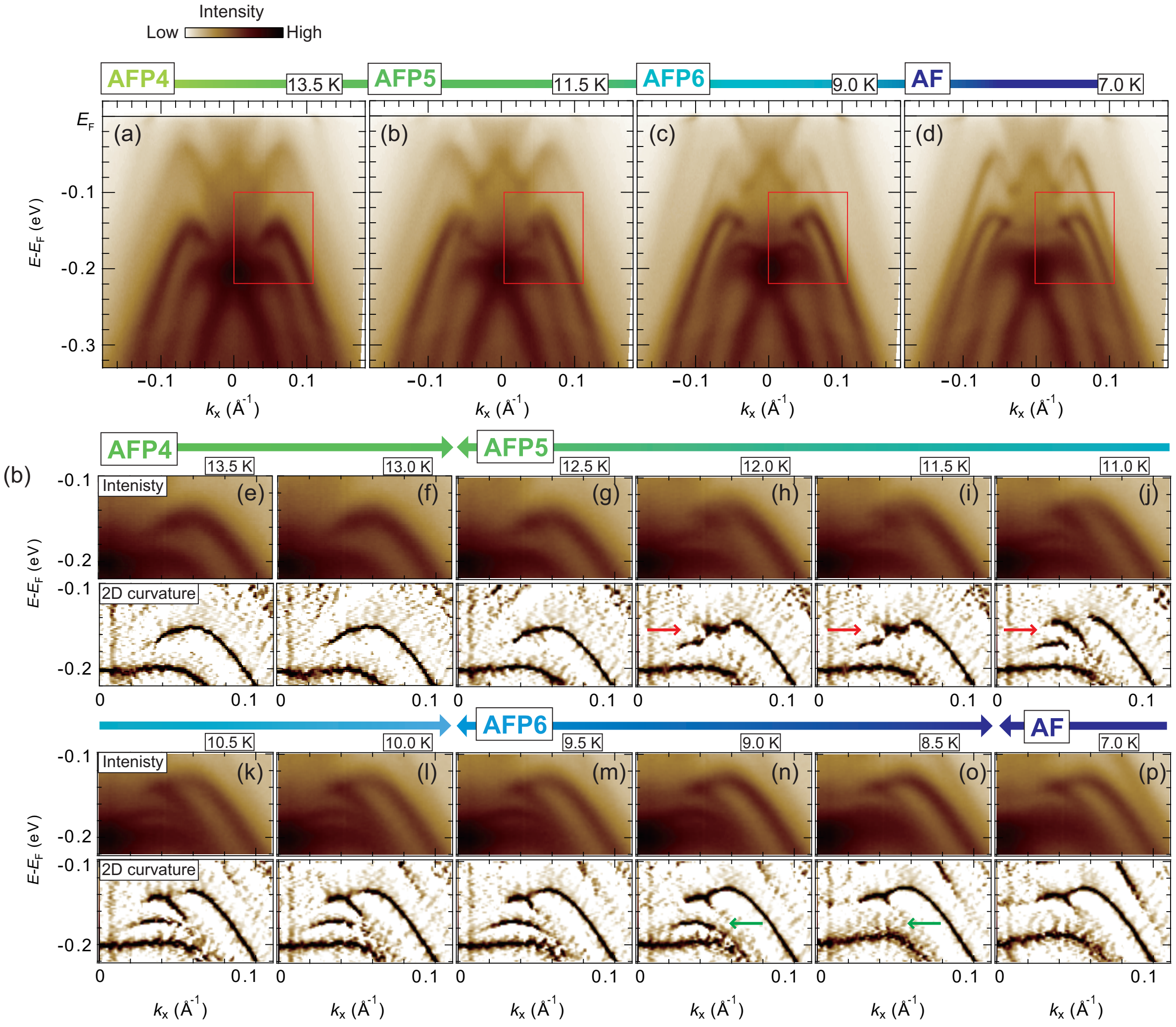

Supplementary Figure 4: **Devilish evolution of the electronic structures.** (a-d) Laser-ARPES images around the E_{F} at the various phases in the devil's staircase evolution. (e-p) The magnified images within the rectangles in (a-d) and their curvature plots to follow the devil's staircase evolution of the band structures. These results display small but clear temperature evolution of the bands at the transitions going from AFP4 to AF phase, highlighted with colored arrows. The M-shaped dispersion is observed in AFP4 phase around $E-E_{\mathrm{F}}=-0.15$ eV [see (a) and (e)], which emerges by the p-d mixing between the main hole and folded bands. It begins to split at the transition to AFP5 phase (red arrows), and is completely changed into three different bands around T=11.0 K [see (j)]. Finally, one of the bands disappears around at the transition to AF phase [green arrows, see (n-p)].

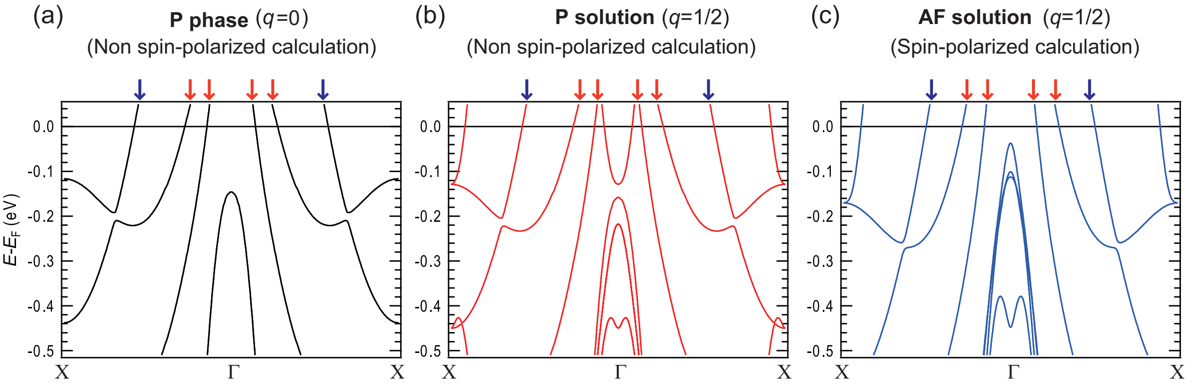

Supplementary Figure 5: **Comparisons of the calculated band dispersions for P and AF phases with non spin-polarized and spin-polarized calculation.** In the main text, we use only the backfolding of paramagnetic (P) bands obtained with DFT+U method with treating $4f$ electrons as core states. Here, in order to show that this is a good approximation to qualitatively reproduce the itinerant band dispersion in AF phase, we have also conducted spin-polarized calculation including $4f$ electrons into valance states (see Methonds), compared to the results with non spin-polarized calculation. (a) The band dispersions for P phase (q=0) obtained with non-spin polarized calculation. (b) With the backfolding of the paramagnetic dispersions (q=1/2), like all of the calculations in the manuscript. (c) The AF band dispersions calculated by spin-polarized calculation including $4f$ electrons. The arrows guide the original hole and electron bands seen in P phase [see (a)]. It is worth mentioning that in DFT+U, we are allowed to apply Hubbard U into only one type of orbital per atom. To make a sensible comparison between spin-polarized and non-spin-polarized, we should also implement Hubbard U into $4f$ electrons for non-spin-polarized. Thus, we cannot additionally apply U into $5d$ electron of Ce in both cases, and consequently, the energy position of the Ce $5d$ band differs from the experimental one [1]. The difference between the results obtained by non spin-polarized calculation (b) and spin-polarized calculation (c) is only to induce hybridizations with all of the folded bands. Accordingly, the most of the bands obtained by the spin-polarized calculation (c) are well consistent with those by the non spin-polarized calculation (b) only consider the backfolding of the paramagnetic bands (a). Thus, the backfolding of the P bands, like we used in the main text, is a good approximation to consider the AF bands.

[1] Kuroda, K. *et al.* Experimental determination of the topological phase diagram in cerium monopnictides. *Phys. Rev. Lett.* **120**, 086402 (2018). URL `https://link.aps.org/doi/10.1103/PhysRevLett.120.086402`.

[2] Strocov, V. Intrinsic accuracy in 3-dimensional photoemission band mapping. *Journal of Electron Spectroscopy and related phenomena* **130**, 65–78 (2003). URL `http://www.sciencedirect.com/science/article/pii/S0368204803000549`.
