# Supplementary material for: Devil's staircase transition of the electronic structures in CeSb
Source: Nat Commun. 2020 Jun 8;11:2888. doi: 10.1038/s41467-020-16707-6 (PMC7280508; doi:10.1038/s41467-020-16707-6)
Supplement: Supplementary file 2 — Description of Additional Supplementary Files [file 41467_2020_16707_MOESM2_ESM.pdf]

Description of Additional Supplementary Files

File Name: Supplementary Movie 1
Description: This supplementary movie records temperature evolutions of laser-ARPES maps for *ab*- and *c*-domain, acquired with changing temperature by a 0.5 K step, and demonstrates the devil's staircase transition of the electronic structures and their spectral responses.)
